# Education and Dementia in the Context of the Cognitive Reserve Hypothesis: A Systematic Review with Meta-Analyses and Qualitative Analyses

**DOI:** 10.1371/journal.pone.0038268

**Published:** 2012-06-04

**Authors:** Xiangfei Meng, Carl D’Arcy

**Affiliations:** 1 Department of Psychiatry, University of Saskatchewan, Saskatoon, Saskatchewan, Canada; 2 Canadian Center for Health and Safety in Agriculture, University of Saskatchewan, Saskatoon, Saskatchewan, Canada; 3 School of Public Health, University of Saskatchewan, Saskatoon, Saskatchewan, Canada; Federal University of Rio de Janeiro, Brazil

## Abstract

**Background:**

Cognitive reserve (CR) or brain reserve capacity explains why individuals with higher IQ, education, or occupational attainment have lower risks of developing dementia, Alzheimer’s disease (AD) or vascular dementia (VaD). The CR hypothesis postulates that CR reduces the prevalence and incidence of AD or VaD. It also hypothesizes that among those who have greater initial cognitive reserve (in contrast to those with less reserve) greater brain pathology occurs before the clinical symptoms of disease becomes manifest. Thus clinical disease onset triggers a faster decline in cognition and function, and increased mortality among those with initial greater cognitive reserve. Disease progression follows distinctly separate pathological and clinical paths. With education as a proxy we use meta-analyses and qualitative analyses to review the evidence for the CR hypothesis.

**Methodology/Principal Findings:**

We searched PubMed, PsycoINFO, EMBASE, HealthStar, and Scopus databases from January 1980 to June 2011 for observational studies with clear criteria for dementia, AD or VaD and education. One hundred and thirty-three articles with a variety of study designs met the inclusion criteria. Prevalence and incidence studies with odds ratios (ORs), relative risks or original data were included in the meta-analyses. Other studies were reviewed qualitatively. The studies covered 437,477 subjects. Prevalence and incidence studies with pooled ORs of 2.61 (95%CI 2.21–3.07) and 1.88 (95%CI 1.51–2.34) respectively, showed low education increased the risk of dementia. Heterogeneity and sensitivity tests confirmed the evidence. Generally, study characteristics had no effect on conclusions. Qualitative analyses also showed the protective effects of higher education on developing dementia and with clinical disease onset hastening a decline in cognition and function, and greater brain pathology.

**Conclusion/Significance:**

This systematic review and meta-analyses covering a wide range of observational studies and diverse settings provides robust support for the CR hypothesis. The CR hypothesis suggests several avenues for dementia prevention.

## Introduction

The Global Burden of Disease Study (GBD) suggests that by 2020 dementia and other neurodegenerative diseases will be the eighth largest source of disease burden in developed countries [1]. The GBD researchers also weighted the severity of disability for a series of health conditions. Out of the ten disorders within the three highest disability classes, eight are neurological problems. Alzheimer’s Disease (AD) is a devastating chronic disease that significantly increases healthcare costs and influences the quality of life of those afflicted and their caregivers. It is the most common degenerative brain disorder. Age at onset varies between 40 and 90 years, in most patients it is after age 65. It is estimated that from 1 to 13% of the population 65 and over have AD [2]–[4]. Vascular disease (VaD) is the second most common dementia disease. Between 1% and 4% of those 65 and over suffer from VaD and the prevalence doubles every 5 to 10 years after the age of 65 [5]. A better understanding of causes of dementia has importance for health researchers and policy makers, in terms of treatment and prevention strategies.

Over the past decades progress has been made in exploration the etiology of dementia. There are many promising leads in the search for the causes(s) of AD. The progressive accumulation and deposition of neurotoxic Aβ/amyloid plaques and tangles that aggregate in the brain with aging–the amyloid hypothesis of AD, is currently a major hypothesis, is likely a necessary but not sufficient cause [6]. The epidemiological literature has found several risk factors for AD including age, gender, education, and ApoE status [7], [8]. Autopsy studies have found that 10% to 40% of individuals with mild to moderate brain pathology did not manifest clinical symptoms of dementia [9], [10]. A hypothetical construct of “cognitive reserve” or “brain reserve” is widely used to explain how, in the face of neurodegenerative changes that are similar in nature and extent, individuals vary considerably in terms of their cognitive decline and clinical manifestation of dementia symptoms [11].

### The Cognitive Reserve Hypothesis

The concept of brain reserve or cognitive reserve (CR) refers to the ability to tolerate the age-related changes and disease related pathology in the brain without developing clinical symptoms or signs of disease [12]. CR also explains the relationship between education, occupational complexity, reading ability, IQ and dementia. This reserve is seen to be a result of changes in the brain itself, resulting from changes in brain structure and processing [13]. Morphological and neurochemical brain changes have been observed in animals raised in stimulating environments [14]. For Stern [15] CR can take two forms: (1) neural reserve in which existing brain networks are more efficient, or have greater capacity, may be less susceptible to disruption; and, (2) neural compensation in which alternate networks may compensate for the pathological disruption of preexisting networks. For Mortimer [16] the fact that pathological lesions can be present long before clinical symptoms of dementia arise suggests that there are two distinct sets of risk factors for AD, “…one for the pathology and the other for clinical expression”. Implicit in the literature is the notion of a threshold which posits that cognitive reserve will limit the clinical expression of the underlying disease until a threshold level of brain pathology is reached at which point the CR can no longer compensate for the underlying physical brain degeneration. Thus the relationship between CR and dementia will differ depending on the underlying pathology [17]. Implicit is the possibility that directly enhancing CR may help forestall the clinical manifestation of AD and dementia [15].

The CR hypothesis postulates that CR acts through both protective and compensatory mechanisms. Individuals with higher levels of CR will have a lower prevalence and incidence of dementia particularly AD. Given that there is both pathology and clinical pathways and if one assumes that the pathological pathway starts at a fixed time in the life course then one would expect that higher CR would lead to a later onset and later death. However if one assumes that there may be a variable time for the start of the pathological process and that process is slow and insidious then the CR hypothesis would suggest that CR will have little or no effect on age of onset of clinical symptoms of dementia and survival (cf. Paradise et al. [18]). Explicit in the CR hypothesis is the notion of a threshold after which CR can no longer compensate for the underlying brain degradation. The CR hypothesis implies that those with higher CR will show a faster cognitive decline after disease symptoms are manifest. It would be consistent with the CR hypothesis to expect those with greater CR would be more sensitive to cognitive impairments and thus expect those with more CR will more likely to present earlier in the clinical progression of the disease and will score higher on initial assessment. Finally it is expected that among those with dementia, individuals with higher CR will show greater brain pathology that those with less CR.

More broadly mental and physical stimulation both early and throughout the life course is thought to increase CR allowing cognitive function to be maintained in old age and to both protect against and delay the onset of dementia and AD.

### This Study

Using education as a proxy measure of CR we explore the evidence linking education and dementia. The CR hypothesis postulates that higher education:

1. reduces the prevalence of dementia;
2. reduces the incidence of dementia;
3. has no affect on the age of onset of dementia;
4. results in an accelerated cognitive decline – as a result of a threshold effect;
5. has no effect on the age of death;
6. leads to a higher level of clinical performance on initial assessment;
7. shows greater brain pathology in postmortem and imaging studies among those with dementia.

We examine the evidence for these hypotheses by systematically review the literature using both meta-analysis and qualitative analysis techniques.

The appropriateness of education as proxy measure has been questioned. Literacy, reading ability or crystallized intelligence have been suggested as better measures of CR [19], [20]. Though far from perfect as a measure of cognitive reserve, education has been measured in a wide variety of studies with diverse populations and methods thus strengthening the rationale for examining its connection to dementia and AD. While formal education is limited as a measure of CR because it is an attribute usually acquired and fixed relatively early in life it may by itself heighten the propensity for greater physical and mental stimulation throughout the life course.

While five review articles published after 2005 have dealt with the relation between education and dementia each has limitations that result in them being incomplete in their review of the research literature [12], [18], [21]–[23]. Fratiglioni and Wang [12] dealt more generally with the CR hypothesis including dimensions other than education and did not use meta-analyses. Two of the review articles, which included meta-analysis contained no more than 20 studies. One article included only case-control and cohort study designs, another just cohort studies and reviewed studies within a narrower time period. Paradise et al. [18] only deal with the relationship between education and survival. The most recent review by Sharp and Gatz [23] is also limited in scope and did not use meta-analyses techniques so no pooled estimates of effect are provided. None of these reviews examined the impact of study quality characteristics on results. As Egger, Smith and Altman point out it is important and desirable to review a body of data systematically, independent of the design and type of study [24].

Our systematic review analyzes data from prevalence and incidence studies with cross-sectional, case-control, and cohort study designs covering a 31-year period. It integrates data from 437,477 subjects and 133 published articles. It includes both a meta-analysis and a qualitative review. In the meta-analysis, the impact of study quality characteristics on results is examined. The qualitative review examines the impact of education on the occurrence of dementia, age of onset, mortality, clinical performance on initial assessment and rate of cognitive decline, and brain degradation is examined. This review fully complies with the PRISMA Statement (see Checklist S1) for systematic reviews and meta-analyses [25].

## Methods

### Search Strategy

We searched the PubMed, PsychINFO, EMBASE, HealthSTAR, and Scopus databases from January 1980 to June 2011 for original research articles in English language. To get maximum number of relevant citations, we employed the followed search strings: ‘(Alzheimer* OR vascular dementia) AND education’ as the keywords for study retrieval.

### Selection of Articles

All suitable articles were evaluated taking into account their internal validity and five selection criterion as follows: 1) have cross-sectional, case-control, or cohort study designs; 2) use clear diagnosis criteria for AD or VaD, specifically DSM and its updates [26], NINCDS-ADRDA [27], ICD-10 [28] or other generally accepted criteria; 3) give clear information on education of study subjects (level of study, years of study); 4) provide a statistical indicator (odds ratio, relative risk, hazard ratio, etc) or original data to estimate the relationship between education and dementia; 5) control potential confounders by using statistical adjustment in the analysis or matching in the study design.

**Figure 1:**
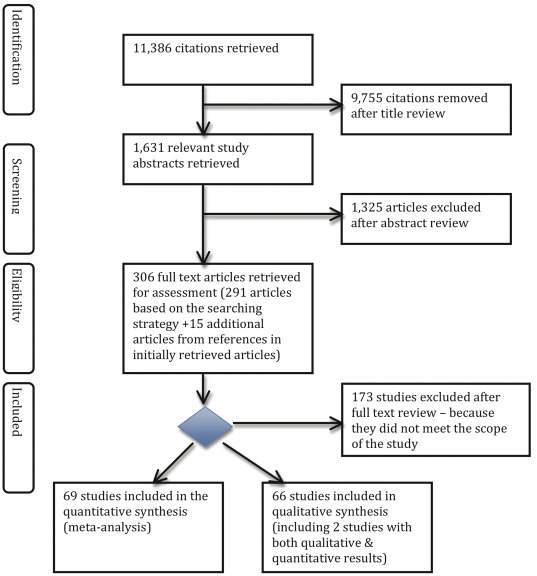
Flow chart of review process.

Figure 1 shows the process of study selection. This initial search produced 11,401 studies. From these studies, 1,631 study abstracts were retrieved for assessment. We also manually searched the references of potential articles that we had retrieved. One hundred and thirty-three articles were included in this systematic review. They are listed in Appendix S1.

### Data Extraction

Data on author, publication year, journal, sample size, methods, indicators, outcomes, adjustments, study design, and results were extracted independently by both authors using a data extraction form. Inconsistencies in interpretation were resolved through discussion. Education was dichotomized into low or high level according to the original article. Disease outcomes were classified based on disease categories reported in the original articles - AD, VaD, and non-specified dementia. Although AD and VaD often co-occur, and can be difficult to be disentangled clinically but become more readily apparent at autopsy. We did not find any reports of mixed dementias in the articles reviewed. The Newcastle Ottawa Scale [29] criteria were used to characterize the quality of case control and cohort studies. Assessment of study quality is essential for a proper understanding of non-randomized studies.

### Data Synthesis

The reviewed articles were divided into quantitative and qualitative studies for further analyses. The quantitative analysis required the studies report data on odds ratio or relative risk or have original study data from which we could calculate an odds ratio. Only quantitative studies were included in the meta-analysis. The qualitative category included studies that reported hazard ratios or whose data on education was not possible to be dichotomized into high and low categories, or which included additional data on age of onset, cognitive decline, clinical performance or mortality. The 133 articles in the final review contained 181 analyses. Sixty-nine studies used in the meta-analysis (quantitative group) and the sixty-six studies used in the qualitative analysis. Two of the quantitative studies also reported qualitative results and are thus used in both the quantitative and qualitative analyses. We report on each category of studies separately.

### Meta-Analysis

The 69 studies reviewed for the meta-analysis included both prevalence and incidence studies. They were used to generate pooled estimates of the effect of education on the prevalence and incidence of the dementias. The studies were stratified as to disease entity – AD, VaD, and non-specified dementia. We separately analyzed the effects of heterogeneity in prevalence and incidence studies. DerSimonian and Laird I^2^ statistics were used to test for heterogeneity [30]. Funnel plots and Egger tests were used to inspect for publication bias [31]. The Egger test provides a more objective way to estimate the reliability of the results. The incidence studies combined show non-significant heterogeneity (Egger test, t = 1.06; *p* = 0.302), but this was not true of prevalence studies (t = 2.29; *p* = 0.025). The later result suggested the possibility of heterogeneity. Thus we choose to use a more conservative random effects model for our analyses. Pooled odds ratios (ORs) were computed by using random effects model. ORs were corrected for publication bias [32]. Sensitivity analysis included the assessment of the influence of each individual study on overall estimates by recalculating ORs with each study being removed one at a time. Sensitivity analysis was also conducted as subgroup analyses removing studies with different types of study designs or different disease outcomes separately. The quality of each study was rated according to the Newcastle-Ottawa Scale and a meta-regression analysis was used to examine the impact of study quality on results. We used STATA, version 8.2, statistical software for the analyses.

## Results

### Quantitative Analysis/Meta-Analysis

Table 1, presents data on the characteristics of the 69 studies included in the meta-analysis. Lead author, date of publication, country in which study took place, education categorization, sample size, study design and crude odds ratios are documented. The studies are grouped as prevalence and incidence studies.

**Table 1:** Education and the risk of dementia: Characteristics of studies included in the meta-analysis.

Study	Country	Education	Sample Size	Study Design	Crude OR (95%CI) AD	VaD	Dementia
**Prevalence (N = 50)**							
Zhang et al. ^[a1]^ 1990	China	≤6 yrs, >6 yrs	5,055	Cross-sectional	9.51 (4.16–21.73)		5.09 (3.03–8.56)
Fratiglioni et al. ^[a2]^ 1991	Sweden	Elementary, High school	1,810	Cross-sectional	1.81 (1.21–2.71)	1.77 (0.98–3.19)	2.08 (1.52–2.83)
Canadian Study of Health and Aging ^[a3]^ 1994	Canada	≤9 yrs, >9 yrs	793	Cross-sectional (base-line)	2.42 (1.74–3.35)		
Kondo et al.^ [a4]^ 1994	Japan	≤Elementary, >Secondary	180	Case-control	1.67 (0.88–3.15)		
Graves et al. ^[a5]^ 1994	USA	≤8 yrs, >9 yrs	1,941	Cross-sectional	64.20 (31.75–129.80)		95.81 (55.33–165.91)
Lobo et al. ^[a6]^ 1995	USA	Primary, Higher	1,080	Case-control	1.12 (0.42–3.01)		1.88 (0.80–4.44)
Liu et al. ^[a7]^ 1995	Taiwan	≤1 yrs, >1 yrs	5,297	Cross-sectional	6.25(0.83–47.01)		
Mortel et al. ^[a8]^ 1995	USA	≤High school, >High school	338	Case-control	1.14 (0.74–1.75)	0.62 (0.38–1.00)	
Ott et al. ^[a9]^ 1995	Rotterdam Netherlands	≤Low vocational training, >Middle level	7,528	Cross-sectional	2.94 (2.32–3.73)	1.44 (0.88–2.38)	1.35 (1.12–1.62)
Callahan et al. ^[a10]^ 1996	USA	≤5 yrs, >6 yrs	2,212	Case-control	3.17 (1.58–6.36)		0.42 (0.24–0.75)
Prencipe et al. ^[a11]^1996	Italy	<3 yrs, ≥3 yrs	1,147	Cross-sectional	2.54 (1.42–4.53)	2.85 (1.20–6.80)	2.74 (1.71–4.39)
Tsolaki et al. ^[a12]]^ 1997	Greece	≤6 yrs, >6 yrs	134	Case-control	0.62 (0.25–1.51)		
Liu et al. ^[a13]^ 1998	Taiwan	Illiterate, Literate	1,736	Cross-sectional	2.97 (1.04–8.46)		3.86 (1.37–10.85)
Lin et al.^[a14]^ 1998	Taiwan	Illiterate, Literate	2,915	Cross-sectional	9.02 (3.26–24.96)	2.07 (0.82–5.20)	2.78 (1.72–4.50)
De Ronchi et al. ^[a15]^ 1998	Italy	Illiterate, Literate	495	Cross-sectional	5.11 (2.36–11.04)		
Hall et al. ^[a16]^ 1998	Nigeria	≤6 yrs, >6 yrs	256	Cross-sectional	0.59 (0.07–5.11)		
* Hall et al. ^[a16]^ 1998	USA	≤6 yrs, >6 yrs	217	Cross-sectional	2.18 (1.02–4.69)		
Harwood et al. ^[a17]^1999	USA	≤10 yrs, >10 yrs	866	Case-control	0.65 (0.45–0.95)		
Hall et al. ^[a18]^ 2000	USA	≤6 yrs, >6 yrs	223	Case-control	1.80 (0.85–3.79)		
Bowirrat et al. ^[a19]^ 2001	Arab	Illiterate, Literate	821	Cross-sectional	9.10 (4.56–18.16)		
Gatz et al.^ [a20]^ 2001	Sweden	≤Elementary, >Elementary	663	Case-control	2.22 (1.04–4.90)		
Bowirrat et al. ^[a21]^ 2002	Israel	Illiterate, Literate	605	Case-control	28.65 (13.79–59.53)	6.32 (2.98–13.42)	
Ravaglia et al. ^[a22]^ 2002	Italy	≤3 yrs, >3 yrs	1,016	Cross-sectional	6.39 (2.71–15.03)	6.77 (2.71–16.94)	6.78 (3.67–12.52)
Lindsay et al. ^[a23]^ 2002	Canada	≤8 yrs, >9 yrs	4,088	Case-control	1.73 (1.28–2.34)		
Mortimer et al. ^[a24]^ 2003	USA	≤16 yrs, >16 yrs	294	Cross-sectional	2.10 (1.05–4.21)		
Harmanci et al. ^[a25]^ 2003	Turkey	≤Primary, >Primary	124	Case-control	2.16 (1.13–4.15)		
Seidler et al. ^[a26]^ 2003	Germany	≤Elementary, >Secondary	424	Case-control	2.09 (1.24–3.51)	2.37 (1.18–4.76)	2.21 (1.43–3.42)
Yu et al. ^[a27]^ 2004	China	Illiterate, Literate	2,674	Cross-sectional	1.08 (0.43–2.70)	0.76 (0.18–3.14)	
Gatz et al. ^[a28]^ 2006	Sweden	Compulsory, Higher	3,373	Case-control	0.96 (0.72–1.28)		2.39 (1.80–3.19)
Zhang et al. ^[a29]^ 2006	China	≤6yrs, >7yrs	34,807	Cross-sectional	2.77 (2.22–3.46)	1.59 (1.19–2.12)	
Zhou et al. ^[a30]^ 2006	China	≤Primary, >Primary	16,095	Cross-sectional	3.05 (2.30–4.03)		
Park et al. ^[a31]^ 2008	Korea	≤Low, >Low	2,187	Cross-sectional	1.93 (1.59–2.33)		
Sahadevan et al. ^[a32]^ 2008	Singapore	≤Primary, >Primary	14,743	Cross-sectional	6.20 (3.14–12.27)	3.76 (1.94–7.31)	4.93 (3.10–7.83)
Fischer et al.^ [a33]^ 2008	Austria	≤Low, >Low	471	Cohort	0.62 (0.37–1.02)		
Grunblatt et al. ^[a34]^ 2009	Germany	≤Secondary, >Secondary	606	Case-control	2.05 (1.34–3.12)		
Gavrila et al. ^[a35]^ 2009	Spain	≤Illiteracy, >Literacy	782	Cross-sectional	2.10 (0.93–4.77)		2.48 (1.38–4.45)
Israeli-Korn ^[a36]^ 2010	Israel	≤Illiteracy, >Literacy	665	Cross-sectional	4.73 (2.97–7.52)		
Mathuranath ^[a37]^ 2010	India	≤4 yrs, >4 yrs		Cross-sectional	1.98 (1.41–2.78)		
Liu et al. ^[a38]^ 1994	Taiwan	≤1 yrs, >1 yrs	455	Cross-sectional			2.88 (0.64–12.83)
Schmand et al. ^[a39]^ 1997	Netherlands	≤Primary, >Primary	4,501	Cross-sectional			2.93 (2.14–4.01)
Herrera et al. ^[a40]^ 2002	Brazil	Illiterate, Literate	1,656	Cross-sectional			2.94 (2.01–4.31)
Zou et al. ^[a41]^ 2003	China	Illiterate, Literate	1,219	Cross-sectional			1.74 (1.12–2.71)
Kahana et al. ^[a42]^ 2003	Israel	≤8 yrs, >8 yrs	1,501	Cross-sectional			2.47 (1.65–3.69)
Ampuero et al. ^[a43]^ 2008	Spain	≤Illiterate, >Literate	359	Case-control			2.20 (1.23–3.95)
Llibre Rodríguez et al. ^[a44]^ 2008	Cuba	≤Primary, >Primary	2,936	Cross-sectional			2.01 (1.44–2.80)
Yamada et al. ^[a45]^2009	Japan	≤7 yrs, >7 yrs	2,105	Cross-sectional			2.48 (1.68–3.66)
Bickel et al.^ [a46]^ 2009	Germany	≤8 yrs, >8 yrs	442	Cross-sectional			4.02 (2.53–6.38)
Arslantas et al.^ [a47]^ 2009	Turkey	≤Illiterate, >Literate	262	Cross-sectional			3.67 (1.47–9.19)
Nunes et al.^ [a48]^ 2010	Portugal	≤Illiterate, >Literate	1,146	Cross-sectional			1.75 (0.80–3.86)
Saldanha et al.^ [a49]^ 2010	India	≤9 yrs, >9 yrs	2,071	Cross-sectional			2.76 (1.00–7.61)
Yaffe et al.^ [a50]^ 2011	USA	≤9 yrs, >9 yrs	998	Case-control			1.99 (1.34–2.97)
**Incidence (N = 22)**							
Beard et al. ^[a51]^ 1992	USA	≤9 yrs, >9 yrs	298	Case-control	0.89 (0.55–1.44)		
Stern et al. ^[a52]^ 1994	USA	≤8 yrs, >9 yrs	593	Cohort	2.32 (1.51–3.58)		
Cobb et al.^ [a53]^ 1995	USA	><High, ≥High	3,330	Cohort	1.36 (0.98–1.89)		
Evans et al.^ [a54]^ 1997	USA	≤8 yrs, >9 yrs	642	Cohort	3.28 (2.00–5.37)		
Zhang et al. ^[a55]^ 1998	China	Illiterate, Literate	1,970	Cohort	2.29 (1.57–3.36)		
Geerlings et al. ^[a56]^ 1999	Netherlands	Low, High	3,778	Cohort	2.80 (1.75–4.46)		
Launer et al. ^[a57]^ 1999	Europe	≤8 yrs, >9 yrs	13,205	Cohort	1.40 (1.12–1.74)		
Letenneur et al. ^[a58]^ 2000	Europe	≤7 yrs, >7 yrs	12,945	Cohort	1.74 (1.40–2.17)		
He et al. ^[a59]^ 2000	China	Illiterate, Literate	2,975	Cohort	3.28 (2.23–5.14)		
* He et al. ^[a59]^ 2000	China	Illiterate, Literate	1,160	Cohort	3.20 (1.99–5.13)		
Kawas et al. ^[a60]^ 2000	USA	≤12 yrs, >12 yrs	1,236	Cohort	0.71 (0.34–1.48)		
* Gatz et al. ^[a20]^ 2001	Sweden	≤6 yrs, >6 yrs	231	Case-control	2.17 (0.69–6.86)		
Qiu et al. ^[a61]^ 2001	Sweden	≤8 yrs, >9 yrs	1,296	Cohort	3.21 (1.97–5.24)		
Karp et al. ^[a62]^ 2004	Sweden	≤7 yrs, >7 yrs	931	Cohort	0.29 (0.16–0.52)		
Yip et al. ^[a63]^ 2006	UK	≤9 yrs, >9 yrs	4,075	Case-control	1.26(0.96–1.64)		
McDowell et al. ^[a64]^ 2007	Canada	≤8 yrs, >9 yrs	6,646	Cohort	3.83 (3.16–4.63)	2.95 (2.14–4.07)	
Lindsay et al. ^[a65]^ 1997	Canada	≤9 yrs, >9 yrs	659	Nested Case-control		2.44 (1.60–3.72)	
Yang et al. ^[a66]^ 2007	China	≤6 yrs, >6 yrs	403	Cohort		2.75 (1.69–4.50)	
* Schmand et al. ^[a39]^ 1997	Netherlands	≤Primary, >Primary	2,176	Cohort			1.86 (1.32–2.63)
Solfrizzi et al. ^[a67]^ 2004	Italy	≤3 yrs, >3 yrs	1,524	Cohort			1.31 (0.38–4.55)
Ngandu et al. ^[a68]^ 2007	USA	≤6 yrs, >6 yrs	591	Cohort			1.77 (0.91–3.41)
Rusanen et al. ^[a69]^ 2011	Finland	≤9 yrs, >9 yrs	20,938	Cohort			1.29 (1.17–1.43)

#### Prevalence studies

**Figure 2:**
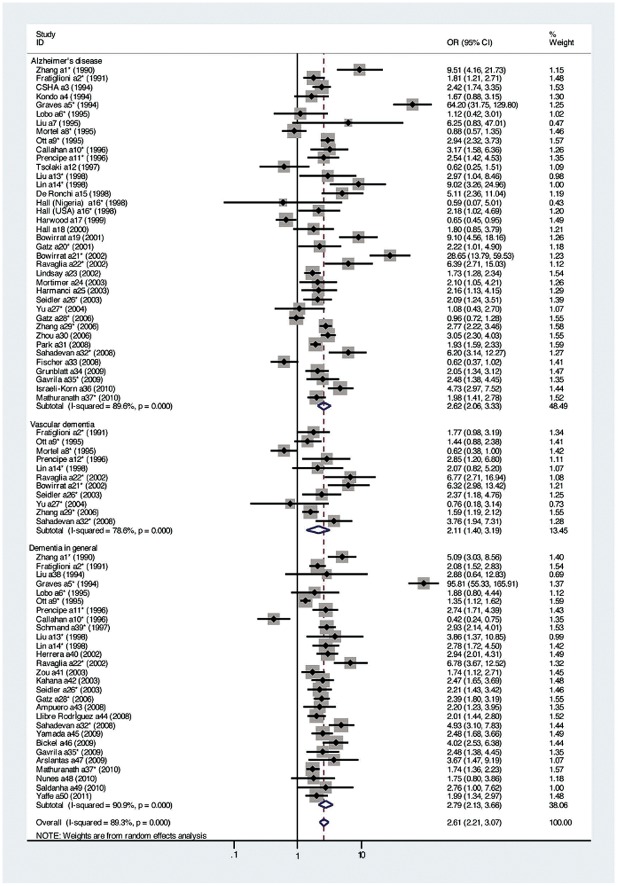
Summary of findings from observational studies of education and prevalence of dementia (* denotes studies with two or more empirical findings).

Figure 2 presents the individual study and pooled estimates for prevalence studies on low education as a risk factor for dementia. The studies are grouped as to disease outcome – AD, VaD, and unspecified dementia. Odds ratio for dementia risk and 95% confident interval are provided. A random effects model was used. Analysis using a fixed effects model did not affect the results.

The pooled OR overall for any dementia for individuals with low education level compared to with high education was 2.61 (95%CI 2.21–3.07, *p*<0.001), indicating that those with low education were 1.61 times more likely to have dementia than individuals with high education. Heterogeneity in this analysis was significant (χ^2^ = 707.08, *p*<0.001). For AD separately, the OR was 2.62 (95%CI 2.06–3.33, *p*<0.001), and the heterogeneity was significant (χ^2^ = 356.74, *p*<0.001). For VaD, the OR was 2.11 (95%CI 1.40–3.19, *p*<0.001), and the heterogeneity was also significant (χ^2^ = 46.81, *p*<0.001). The OR for unspecified dementia was 2.79 (95%CI 2.13–3.66, *p*<0.001), and the heterogeneity was also significant (χ^2^ = 295.99, *p*<0.001).

#### Incidence studies

**Figure 3:**
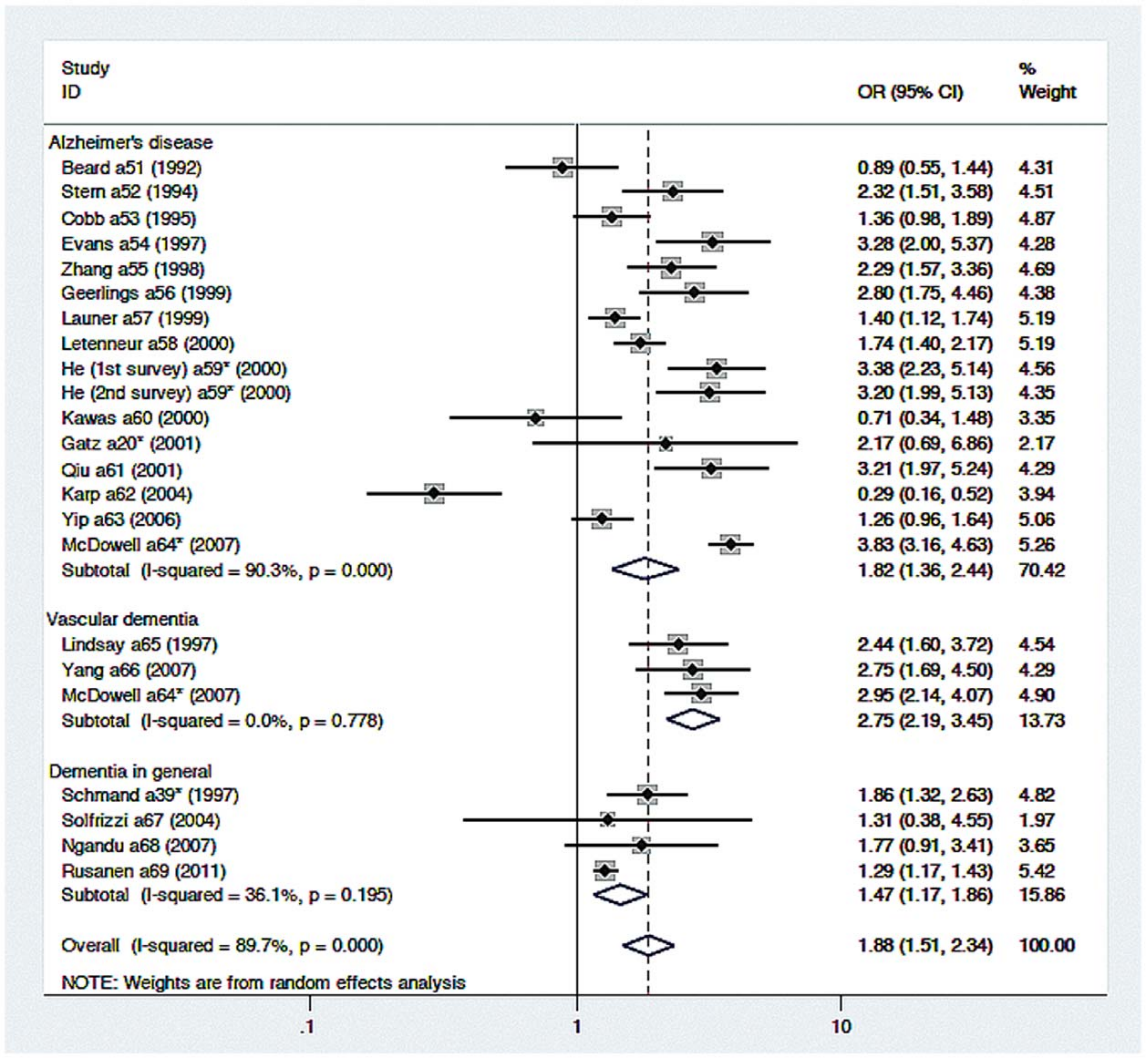
Summary of findings from observational studies of education and the incidence of dementia (* denotes studies with two or more empirical findings).

Figure 3 shows the individual study and pooled estimates for incidence studies. The meta analysis used a random effects model, and results were unchanged using fixed effects model. The pooled OR for individuals with low education level compared to with high education was 1.88 (95%CI 1.51–2.34 *p*<0.001), indicating low education level were 0.88 times more likely to develop dementia than individuals with high levels of education. Heterogeneity here was significant (χ^2^ = 213.60, *p*<0.001). In these incidence studies the low/high education OR for AD was 1.82 (95%CI 1.36–2.44, *p*<0.001), heterogeneity was also significant (χ^2^ = 155.03, *p*<0.001). For VaD in these studies the low/high OR was 2.75 (95%CI 2.20–3.45, *p*<0.001) while heterogeneity was not significant (χ^2^ = 0.50, *p* = 0.78). The incidence OR for unspecified dementia was 1.48 (95%CI 1.17–.86, *p*<0.001), and the heterogeneity was not significant (χ^2^ = 4.70, *p* = 0.195).

#### Publication bias

**Figure 4:**
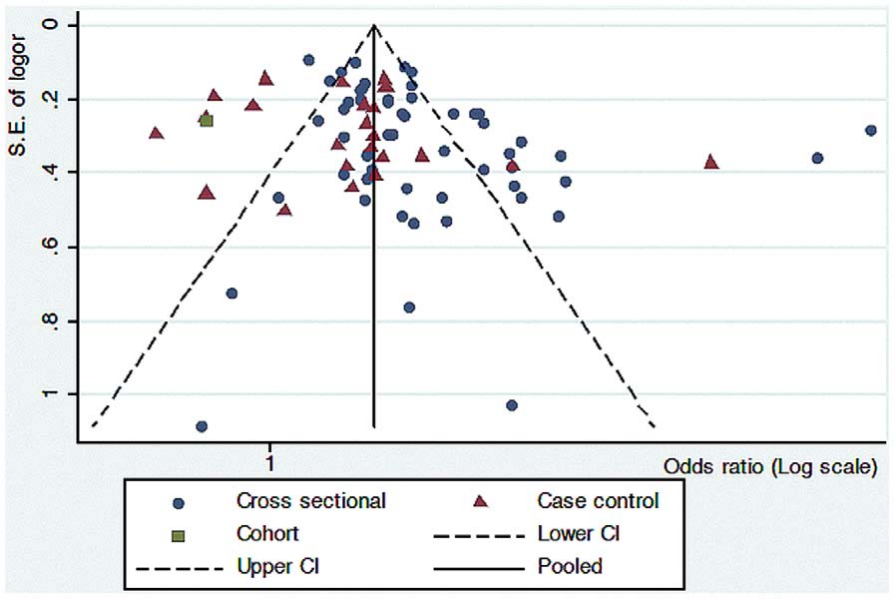
Funnel plot prevalent studies of dementia and education with a variety of study designs.

**Figure 5:**
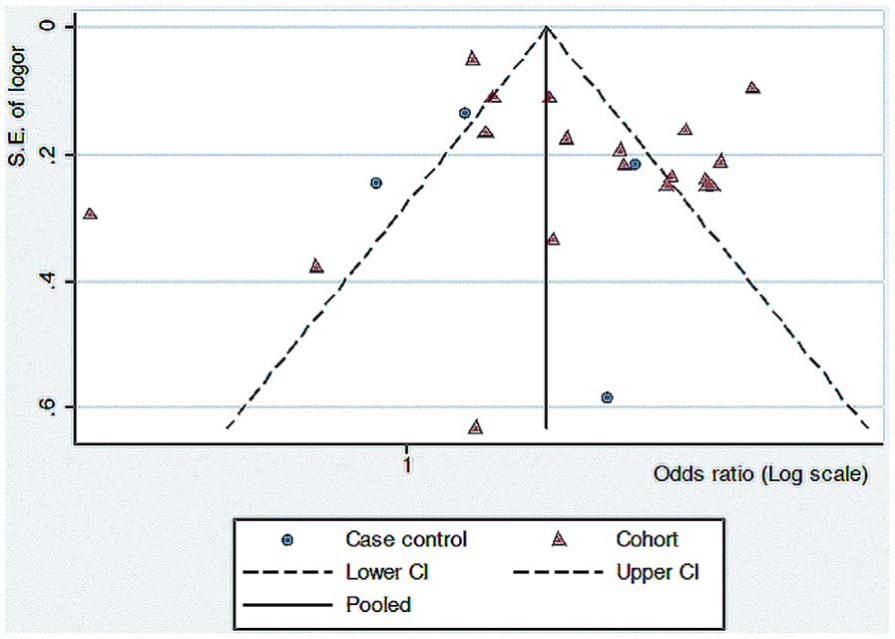
Funnel plot of incidence studies of dementia and education.

Funnel plots were used to visually assess the presence of publication bias. As shown in Figures 4 (prevalence studies) and Figure 5 (incidence studies), most of studies are within the domain, which represent 95% confidence interval limits around the estimate, though some articles exceed these boundaries. In these plots the X-axis shows the logarithmic scale of odds ratio estimate for each study and Y-axis is standard error of the logarithmic function of the odds ratio. The dashed line represents the 95% confidence intervals and the point estimate of logarithmic transition of odds ratio illustrates as the solid line. No apparent asymmetry is shown in these funnel plots.

To examine the issue of heterogeneity more objectively, we employed the Egger test separately for different diseases and study designs. There were no significant differences between different diseases in prevalent and incident studies (*P*>0.05). Only for cross sectional (prevalence) studies was the Egger test significant (t = 2.30; *p* = 0.003). We then used the “trim-and-fill” method proposed by Duval and Tweedie [32] to further re-examine publication bias in prevalence studies. The resulting estimate of the corrected OR was 1.87 (95%CI 1.56–2.24, *p*<0.001), while the corrected OR was reduced, it did not change the essential thrust of the previous findings for prevalence studies on the effect of low education being associated with a higher prevalence of dementia.

#### Sensitivity analyses

Sensitivity analyses assessed the influence of each study on overall estimates by omitting one study at a time. For prevalence studies, these analyses yield low/high education ORs ranging from 2.50 (95%CI 2.13–2.93) to 2.66 (95%CI 2.26–3.13); while for incidence studies, the ORs vary from 1.76 (95%CI 1.45–2.14) to 2.03 (95%CI 1.65–2.49). In subgroup analyses both prevalence studies and incidence studies were stratified by study design. The corrected OR for case control prevalence studies was 1.68 (95%CI 1.25–2.26; z = 3.43, *p*<0.001); for cross sectional prevalence studies the corrected OR was 3.23 (95%CI 2.68–3.88; z = 12.39, *p*<0.001); a similarly corrected OR for cohort incidence studies was 1.96 (95%CI 1.54–2.51; z = 5.40, *p*<0.001); the corrected OR for case-control studies incidence studies was not significant at 1.47 (95%CI 0.93–2.34; z = 1.64, *p* = 0.100). This sensitivity analyses generally supports the conclusion that low education is a risk factor for dementia.

#### Meta-regression

We accessed the relationship between study quality and observed risk through meta-regression of the rating of study quality. Generally none of the specific study characteristics examined had an impact of the observed ORs. None of characteristics of case control or cohort studies show significantly independent results (*p*>0.05).

#### Quantitative analyses conclusions

The above meta-analyses covering a wide range of studies and diverse populations provides robust evidence that higher education substantially reduces the prevalence and incidence of AD, VaD and nonspecific dementia. The pooled odds ratios for this large number of studies strongly support the CR hypothesis that postulates higher education:

1. reduces the prevalence of dementia.
2. reduces the incidence of dementia

### Qualitative Analyses

The qualitative analyses reviewed the 66 studies with qualitative data, included are results from 2 studies that also reported quantitative data included in the meta-analysis above. They contain 79 specific results. These studies and findings are annotated in Table 2. Qualitative studies are those studies, which reported hazard ratios or whose data on education was not possible to be dichotomized into high and low categories, or which included additional data on age of onset, cognitive decline, clinical performance or mortality. For reporting purposes we have categorized these studies in terms of the focus of their findings.

**Table 2:** Qualitative analysis: Studies linking education to various aspects of dementia (* denotes studies with findings related to two or more areas of inquiry).

Study	Country	Study Design	Sample Size	Results
**Prevalence (N = 20)**				
Letenneur et al. ^[a70]^ 1999	France	Cohort	2,881age 65+	Subjects with a lower educational level were at higher risk of developing AD.
Yamada et al. ^[a71]^ 1999	Japan	Cohort	2,222 age 60+	More education can protect against AD and VaD.
Tyas et al. ^[a72]^ 2001	Canada	Cohort	694 age 65+	Lower education was significantly related to occurrence of AD.
Anttila et al. ^[a73]^ 2002	Finland	Population Cohort	1,449	Low education and the APOE ε4 allele independently increased the risk for dementia.
Di Carlo et al. ^[a74]^ 2002	Italy	Cohort	2,266 age 65+	A higher level of education was protective against dementia and AD.
Miech et al. ^[a75]^ 2002	USA	Cohort	4,092 age 65+	Education had no association with the incidence of AD.
Wilson et al. ^[a76]^ 2002	USA	Cohort	835 age 65+	Education was inversely associated with risk of AD, but the effect was substantially reduced when cognitive activity was added to the model.
Yamada et al. ^[a77]^ 2003	Japan	Cross-sectional	1,774	AD prevalence increased significantly with lower education.
Ravaglia et al. ^[a78]^ 2005	Italy	Cohort	937 age 65+	A higher level of education was protective against the risk of dementia and AD but not for VaD.
Tognoni et al. ^[a79]^ 2005	Italy	Cross-sectional	1,600 age 65+	Higher education can protect against AD, not VaD.
Lin et al. ^[a80]^ 2006	Taiwan	Case-control	245	There was no relation between education and VaD.
Shadlen et al. ^[a81]^ 2006	USA	Cohort	2,786	Low level education was risk factor of incident AD, and black people have more risk than white people.
van Oijen et al. ^[a82]^ 2007	Netherlands	Cohort	6,927 age 55+	Both men and women with higher education had a lower risk of AD although the association seemed strongest in men.
Galasko et al. ^[a83]^ 2007	Guam	Cross-sectional	2,770 age 65+	Decreasing education was significantly associated with an increased prevalence dementia overall.
Lee et al. ^[a84]^ 2008	Korea	Cohort	966 age 65+	Illiteracy was associated with a higher risk of AD and the risk increases with age.
Yamada et al. ^[a85]^ 2008	Japan	Cohort	2,286 age 60+	Probable AD decreased with increasing education level. Probable VaD showed no significant effects of education.
Hebert et al. ^[a86]^ 2010	USA	Cohort	1,695 age 65+	Incidence of AD did not change over a 3-year period and the relationship between education and AD also did not change. Education was not statistically significantly associated with AD though the odd ratio trended towards a protective effect for higher education.
EClipSE Collaborative Members ^[a87]^ 2010	Europe	Cohort	872	Longer years in education were associated with decreased dementia risk.
Rastas et al. ^[a88]^ 2010	Finland	Cohort	339 age 85+	Higher level of education were associated with a lower probability of dementia.
Kim et al. ^[a89]^ 2011	Korea	Cross-sectional	1,673 age 65+	Lower education level was associated with a higher risk of the onset of dementia.
**Incidence (N = 2)**				
*Tuokko et al.^ [a98]^ 2003	Canada	Cohort with nested case-control sub-study (CSHA)	844 aged 65+	Subjects were classified as high (HF) and low (LF) functioning in three ways: education, occupation, and estimated premorbid IQ. Fewer HF adults were diagnosed as incident dementia after 5 years.
*Tervo et al. ^[a100]^ 2004	Finland	Cohort	806 age 60+	Over a 3-yr study period those with high education were less likely to convert to mild cognitive impairment (MCI), a potential precursor of AD, than those with low or no education.
**Cognitive Decline (N = 20)**				
Filley et al. ^[a90]^ 1985	USA	Case-control	28	Higher education did not delay the onset of AD or Senile Dementia of the Alzhiemer’s Type (SDAT) nor did it retard the rate of intellectual deterioration or functional decline.
Teri et al.^ [a91]^ 1995	USA	Cohort	156, age 65+	Higher education was significantly related to increasing rates of cognitive decline.
Small et al. ^[a92]^ 1997	Sweden	Cohort	36	There was no relation between education and cognitive decline.
Aguero-Torres et al. ^[a93]^ 1998	Sweden	Cohort	243 age 75+	Education did not affect rates of cognitive decline.
Weiner et al. ^[a94]^ 1998	USA	Cohort	594	Persons with completed high school education in contrast to those who had not completed high school had a more rapid decline on cognitive function, language and praxis scales but no greater loss in global function.
Stern et al.^ [a95]^ 1999	USA	Cohort	177	There was a more rapid decline in memory scores in AD patients with high educational and higher occupational attainment especially among those with low initial scores.
Wilson et al. ^[a96]^ 2000	USA	Cohort	410	Higher educational attainment was associated with higher baseline cognitive function and more rapid decline.
*Bowirrat et al. ^[a21]^ 2002	Israel	Case-control	285age 60+ (Arab population)	Illiteracy was significantly more common among those who developed AD than among those who remained with Age Associated Cognitive Decline (ARCD).
Frittsch et al. ^[a97]^ 2002	USA	Cohort	482	Higher education slowed the rate of cognitive decline in persons with AD.
Tuokko et al.^ [a98]^ 2003	Canada	Cohort with nested case-control sub-study (CSHA)	844 aged 65+	Subjects were classified as high (HF) and low (LF) functioning in three ways: education, occupation, and estimated premorbid IQ. Those HFs diagnosed with dementia showed more rapid decline on 5 of 6 measures memory measure; they also had lower baseline scores than those HFs who did not become demented.
Suh et al.^ [a99]^ 2004	South Korea	Cohort	107 community based AD patients	Neither gender, duration of education, nor duration of AD since onset were significant predictors of cognitive and functional decline.
Tervo et al. ^[a100]^ 2004	Finland	Cohort	806 age 60+	Over a 3-yr study period those with high education were less likely to convert to mild cognitive impairment (MCI), a potential precursor of AD, than those with low or no education.
Wilson et al. ^[a101]^ 2004	USA (Chicago)	Cohort	494AD patients	Higher education level was related to more rapid global cognitive decline with education related to the non-linear but not the linear component of the decline. Age was related to linear decline, with more rapid decline observed in younger persons.
Le Carret et al. ^[a102]^ 2004	France	Case-Control	20 Cases, 20 Controls	Higher educated (HE) patients exhibited greater impairment of abstract thinking whereas low-educated patients showed greater impairment of memory and attention. Some cognitive processes such as abstract thinking may decline more rapidly in HE but they may still benefit from cognitive reserve even after a dementia diagnosis.
Andel et al. ^[a103]^ 2006	USA	Cohort	171 AD patients	High education, high substantive complexity, and high complexity of work with data and people predicted faster rates of cognitive decline controlling other attributes. Cognitive reserve may postpone the clinical onset of AD but accelerate cognitive decline after the onset.
Hall et al. ^[a104]^ 2007	USA	Cohort	117 age 75∼85	Higher education delayed the onset of accelerated cognitive decline; once it started it was more rapid in persons with more education.
Bruandet et al. ^[a105]^ 2008	France	Cohort	670	Highly educated patients had a faster cognitive decline than less educated patients.
Helzner et al. ^[a106]^ 2009	USA	Cohort	156 mean age 83	Highly educated patients had a faster cognitive decline than less educated patients.
Musicco et al. ^[a107]^ 2009	Italy	Cohort	154	More educated persons were more likely to have faster Alzheimer’s disease progression.
Chaves et al. ^[a108]^ 2010	Brazil	Cohort	80	More education was a strong predicator for faster cognitive decline.
**Age of Onset (N = 8)**				
*Filley et al. ^[a90]^ 1985	USA	Case-control	28	Higher education did not delay the onset of AD or Senile Dementia of Alzheimer Type (SDAT).
Montz et al. ^[a109]^ 1993	USA	Cross-sectional	1,658	Reported age of onset was later among those with less education but clinically rated severity of disease was a greater among those with less education. Authors suggest low education may lead to later detection and referral for treatment.
*Bowler et al. ^[a122]^ 1998	Canada	Cohort	172 AD patients	Higher education had a modest effect on earlier reported onset of AD which probably reflecting earlier recognition.
*Weiner et al. ^[a94]^ 1998	USA	Cohort s	594	Persons with completed high school education in contrast to those who has not completed high school present clinically at a significantly earlier age.
*Del Ser et al. ^[a125]^ 1999	Canada	Cohort	87 AD patients	Less education patients became demented later.
Pai et al. ^[a110]^ 2002	Taiwan	Case-control	155	Education level was not related to the onset age of AD.
Roe et al. ^[a111]^ 2008	USA	Case series.	23,329	Reported age of on set of dementia symptoms is slightly younger among those with more education.
Lupton et al. ^[a112]^ 2010	UK	Cohort	1,320	Education did not delay the onset of AD.
**Mortality (N = 8)**				
Stern et al. ^[a113]^ 1995	USA	Cohort	246	Patients with more education had increased mortality.
Geerlings et al. ^[a114]^ 1997	USA	Cohort	4,022 age 65+	Higher education was not associated with increased risk of mortality in AD patients.
*Aguero-Torres et al. ^[a93]^ 1998	Sweden	Cohort	243 age 75+	Less educated subjects had significantly shorter survival.
*Del Ser et al. ^[a123]^ 1999	Canada	Cohort	87 AD patients	Less education patients became demented later and died later.
*Qiu et al. ^[a61]^ 2001	Sweden	Cohort	1,296 age 75+	A low educational level was not related to the mortality of subjects with AD or dementia.
Brehaut et al. ^[a115]^ 2004	Canada	Cohort	9,681 age 65+	For subjects with no cognitive impairment, higher education was associated with lower mortality, while among cognitively impaired elderly, there was no association between education and mortality.
Pavlik et al. ^[a116]^ 2006	USA	Cohort	478	Education was not associated with survival.
*Bruandet et al. ^[a105]^ 2008	France	Cohort	670	Highly educated and less educated patients had similar mortality rates.
**Clinical Performance (N = 6)**				
*Weiner et al. ^[a94]^ 1998	USA	Cohort	594	Persons with completed high school education presented clinically with higher cognitive scores than persons with high school incomplete.
Swanwick et al. ^[a117]^ 1999	Ireland	Case-control	209	Patients with only a primary-school education had a trend towards lower cognitive scores at presentation but did not have more functional deficits.
*Wilson et al. ^[a96]^ 2000	USA	Cohort	410	Higher educational attainment was associated with higher baseline cognitive function.
Ott et al. ^[a118]^ 2008	USA	Case-control	128	Lower education was associated with worse performance.
*Roe et al. ^[a111]^ 2008	USA	Case series.	23,329	Participants with fewer years of education show greater clinical severity of AD at first assessment. Symptoms of AD are recognized later by those with less education.
Aguera-Ortiz et al. ^[a119]^ 2010	Spain	Cohort	1,235 mean age 77.8	Education has shown to be beneficial in delaying the clinical manifestation of AD.
**Pathology and Imaging (N = 15)**				
Stern et al. ^[a120]^ 1992	USA	Case-control	58	Cerebral blood flows were comparable in 3 groups of patients with varying levels of education. However parietotemporal perfusion deficit was significantly greater in the groups with the highest level of education indicating that AD was more advanced in this group.
Kidron et al.^ [a121]^ 1997	Canada	Case-control	32 AD patients and 20 age and gender matched healthy controls	Computerized imaging analysis techniques were applied to MR brain images. More years of education had a direct effect on parietal atrophy. Education was seen to have a tardive affect on the clinical evolution of AD.
Bowler et al. ^[a122]^ 1998	Canada	Cohort	172 AD patients	The outcome measures were age of onset, cognitive decline and post-mortem pathology. Education level, occupation level, gender, family history, year of birth, age of onset, severity of disease at entry, ischemic score, and the presence of leukouriosis, did not affect age of onset or rate of cognitive decline.
Del Ser et al. ^[a123]^ 1999	Canada	Cohort	87 AD patients	Cognitive function declined at the same rate in all educational groups and there was no difference in neurodegenerative lesions.
Zubenko et al.^ [a124]^ 1999	USA	Cohort	330 asymptomatic 1st degree relatives of probands with AD	Age increased platelet membrane fluidity and the APOE ?4 allele made significant independent contribution to the risk of developing AD while gender and years of education did not.
Munoz et al. ^[a125]^ 2000	Canada	Case-control	267	The educational attainment for patients with autopsy-confirmed AD was no different from that of a sample of patients who underwent autopsies at the same hospital and in whom the autopsy did not show neurodegenerative disease. Occupation and income were not statistically different between the two groups.
Bennett et al.^ [a126]^ 2003	USA	Cohort	130 older Catholic clergy	Education modified the relationship of neuritic plaques and diffuse plaques to cognition but not neurofibrillary tangles. The interaction between education and neuritic plaque score was strongest for perceptual speed and weakest for episodic memory. The relation between senile plaques and level of cognition differed by years of formal education.
Liao et al.^ [a127]^ 2005	USA	Case-control	132	Years of formal schooling had negative associations with cerebral perfusion. The authors suggest the main effect of more education is a more facile use of alternative brain circuits instead of locally increased synaptic connections.
Mortimer et al. ^[a128]^ 2005	USA	Cohort	294 Catholic sisters (the Nun Study)	Among those Catholic sisters with high (vs low) educational attainment the frequency of the clinical diagnosis of dementia before death was reduced by 26% for those with milder neuropathologically autopsy confirmed states of the disease (Braak stage 1–111) and 13% for those with more severe pathology (Braak stages IV–VI). Education has a powerful effect in reducing the clinical severity of dementia individuals with moderate AD pathology. As severity of pathology increases the protective role of education remains but is diminished. It is insufficient to mask the presence of substantial brain pathology.
Perneczky et al.^ [a129]^ 2006	Germany	Case-control	93 consecutive patients with mild AD and 16 age-matched healthy controls	There was a marked inverse association between years of schooling and glucose metabolism and glucose metabolism in the posterior temporo-occipital association cortex and the precuneus in the left hemisphere. Findings suggest that education is associated with brain reserve and those with higher education can cope with brain damage for a longer time.
Roe et al. ^[a130]^ 2007	USA	Cohort	2,372 age 65+	Subjects with neuropathologically diagnosed AD who had more years of education are less likely to be diagnosed as demented at last clinical assessment that occurred within one year prior to death. Those clinically diagnosed as demented had an average 2 to 3 years less education than the clinical non-demented group. Individuals with more education seem able to withstand greater amounts AD-associated brain damage.
Hanyu et al.^ [a131]^ 2008	Japan	Cohort	53 AD patients	Initially the high education group (HE ≥12 years of schooling) had greater regional cerebral blood flow (rCBF) deficits even though there were no differences between education groups on cognitive and functional assessment scores. The HE group demonstrated more extensive and severe reduction of rCBF on follow-up Single Photon Emission Computed Tomography (SPECT) in association with faster cognitive and functional decline.
Koepsell et al. ^[a132]^ 2008	USA	Cohort	2,051 age 65+	When there was no evidence of post-mortem neuropathology or when the neuropathology was mild, higher education was associated with higher MMSE score. When AD neuropathology was more advanced educational differences in MMSE score became more attenuated.
*EClipSE Collaborative Members^ [a87]^ 2010	Europe	Cohort	872	More education did not protect individuals from developing neurodegenerative and vascular neuropathology by the time they died but it did appear to mitigate the impact of pathology on the clinical expression of dementia before death.
Cordonnier et al. ^[a133]^ 2010	France	Cohort	417 Pre-existing dementia in patients with intracerebral haemorrhages	In lobar intracerebral haemorrhage, factors associated with pre-existing dementia were increasing age, low educational level and severity of atrophy suggested a neurodegenerative process this contrasted with the findings for patients who had deep intracerebral haemorrhage. Autpsied results suggests dementia is these patients is a result of two different neurodegeneration and vascular mechanisms.

#### Prevalence studies

Of the 20 qualitative prevalence studies reviewed 18 (90%) reported that higher education was a protective factor with higher education decreasing the risk of AD. The remaining studies reported non-significant trend in that direction. The 6 studies that had data on VaD prevalence split with 3 studies reported higher education was protective and 3 reported no educational effect.

#### Incidence studies

Both studies in this category found that increasing education was protective against the onset of dementia and AD.

#### Cognitive decline studies

Twenty studies reported on cognitive decline. The majority, 14 (70%), found that higher education lead to a more rapid cognitive decline. Only 2 studies (10%) reported slower decline among those with higher education while in 4 studies (20%) education was found to have no effect on rate of cognitive decline. More recent studies from 2004 onwards almost uniformly report a more rapid decline. Three of the more recent studies note a threshold effect with those with higher education having an initial slower decline that then accelerates.

#### Age at onset studies

Five of the 8 (62.5%) studies with age of onset data report that those with lower education reported a later age of onset consequently higher education was associated with earlier age of onset. The remaining three studies failed to detect any association.

#### Mortality studies

Educational difference in mortality were not found in 5 of 8 (62.5%) studies; 2 studies found those with higher education died earlier and the remaining study reported those with less education died earlier. Paradise et al’s meta-analysis also found more education did not lead to earlier death after diagnosis [18].

#### Initial clinical assessment studies

These studies almost uniformly (7 of 8) found those with higher education had higher cognitive performance at initial assessment.

#### Pathology and imaging studies

Seventy percent of studies (10 of 14) report increased pathology and degradation in brain function among those with more education. In general these studies support the notion that those with education are able to deal with great brain pathology before the clinical manifestation of the underlying disease becomes apparent. It is of interested to note that 3 of the 4 studies that report no relationship between brain pathology and education come from essentially the same research center.

#### Descriptive analyses conclusion

These studies are generally supportive of the CR hypothesis that postulates higher education:

1. has no effect on the age of onset of dementia
2. after onset of clinical symptoms, results in an accelerated cognitive decline –a threshold effect is implied
3. has no effect on the age of death
4. leads to a higher level of clinical performance on initial assessment
5. shows greater brain pathology in postmortem and imaging studies among those with dementia.

## Discussion

### Findings

The meta-analyses of quantitative studies reported on here strongly and consistently show that those with lower education had a higher risk for dementia, the pooled OR was 2.61 (95%CI 2.21–3.07) for prevalence studies and 1.88 (95%CI 1.51–2.34) for incidence studies, providing robust evidence that a higher level education resulted in a significant reduction both in prevalence and incidence dementia, either AD, VaD or unspecified dementia. The analyses of descriptive studies also firmly supports the CR hypothesis that postulates that people with higher education will have a lower prevalence and incidence of dementia, higher scores at initial assessment; higher education has no affect on the age of onset, after the onset of clinical symptoms results in an accelerated cognitive decline, and has no effect on the age of death. The biological studies reviewed also show that those with higher education had greater brain damage; but that damage did not translate initially into poorer cognitive performance. There is both clinical and biological support for a ‘threshold effect’ where higher education initially masks the clinical manifestation of dementia but after brain pathology reaches a critical level there is a more rapid cognitive decline.

### Strength of Present Review

Our meta-analysis was based on data from over 437,477 subjects from a wide range of countries materially surpassing previous reviews [12], [20], [21]. We also include an analysis of descriptive studies that cover dimensions of the CR hypothesis other than incidence and prevalence.

### Limitations-Quantitative Analytic Issues

Though there is some continuing debate on the merit of quantitative meta-analysis of observational reports [33], there is a need to evaluate the reliability and validity of observational study results when experimental methods (randomized controlled trials) are clearly inappropriate, impossible, or inadequate [34].

The present systematic review clearly shows that, people with low education are more likely to suffer from dementia. This is a robust finding evident in both prevalence and incidence studies and in a wide range of settings. The ORs are still significantly greater than 1.0 whether stratified by disease subgroups or adjusted for 8 study quality indicators.

Heterogeneity is a concern in the meta-analysis of observational studies. It can be clinical and/or statistical heterogeneity. Funnel plots of both prevalence and incidence studies give an indication of the possibility of heterogeneity existing. The assessment of the heterogeneity in meta-analysis is a crucial issue because the presence versus the absence of true heterogeneity (between study variability) can affect the statistical model that the meta-analyst decides to apply to the meta-analytic database [35]. The sources of heterogeneity can be reporting bias, which includes publication bias, language bias, citation bias, and multiple publication bias, true heterogeneity (which shows effect sizes differ according to study size) intensity of intervention and differences in underlying risk, data irregularities (which can result from poor methodological design of small studies) inadequate analysis, and fraud, and chance [36].

Data for our systematic review and meta-analyses was based on searches of 5 major databases covering a 31-year period. Our retrieving language restriction may evoke the suggestion of a language bias. However as English is more and more frequently used in scientific discourse the probability of language bias becomes less likely. Given our study collection and extraction procedures it is much more likely than any bias that may arise would be a result of our analysis procedures.

Our analysis of the influence any individual study shows no substantial difference in pooled ORs when individual studies are omitted. This analysis was necessary because of the number of studies included in the review. None of prevalence studies or incidence studies reviewed reported negative significances. Differences in the design of observational studies are a major source of heterogeneity. When stratified by study design in subgroup analyses, only case control incidence studies had non-significant findings. There were only 4 articles included as case control incidence studies. The small number of studies is likely to produce unstable conclusions. Since the Egger’s heterogeneity test on cross sectional prevalence studies was significant, the “trim and fill” method was used to explore the influence of potential publication bias in prevalent studies. The resulting corrected OR did not change our conclusion that, low education is a risk factor for dementia.

It is notable that both prevalence and incidence studies showed positive evidence that high education is a protective factor of against dementia. Low education is not merely a risk factor for dementia, but is a contributory factor in the development of dementia. The findings of this systematic review support the general tenets of the cognitive reserve hypothesis [12], [15]–[16], [20]–[21].

### Limitations-Qualitative Analytic Issues

Articles reviewed qualitatively reported hazard ratios or data on education that we were not able to dichotomize into high and low categories, or included additional data on age of onset, cognitive decline, clinical performance or mortality, and brain pathology. It was not possible to include those studies in the meta-analysis section of this review. However, to further our understanding of the role of education in dementia, a qualitative analyses of these studies was undertaken. The picture that emerges from the qualitative analysis is that higher education is generally protective against the onset of dementia, particularly AD. Those with higher education generally presented clinically earlier in the progression of disease, have higher clinical scores at presentation, and decline slowly in cognitive function initially but after a threshold level has been breached a more rapid decline ensues. Biological studies indicate that subjects with more formal schooling had greater brain damage but that damage did not translate initially into poorer cognitive performance. These qualitative studies were supportive of the CR hypothesis.

### Answered and Unanswered Questions

The findings in this systematic review both the quantitative and qualitative, are consistent with the CR hypothesis in which early education and stimulation may be seen to affect brain structure [12]. Extensive social networks, active engagement, or regular participation intellectually stimulating activities significantly lower the risk of AD and other forms of dementia. In addition to the CR hypothesis, the ‘brain battering’ hypothesis, which presumes that less educated individuals’ increased risk of dementia are related to increased vascular lesions, may also be invoked to explain the association between education and dementia [37]. However such a hypothesis is inconsistent with the post-mortem, imaging and cellular activity studies, which show greater brain pathology among those with higher education. Further research is needed to elucidate the underlying biological mechanisms by which education protects against the onset of dementia and influences its course and outcome.

### Conclusions

The present study demonstrates robust evidence that a high level education in early life is related with a significant reduction both in the prevalence and incidence of dementia, including AD and VaD. These results are in accordance with the CR hypothesis, which assumes some aspects of life experience such as education protects against the onset of dementia. Education also influences the course and outcome of the disease in terms the pattern of cognitive decline and underlying brain pathology.

Study findings that adult-life work complexity, social network and complex leisure activities also reduce the occurrence of dementia complements the evidence presented in this systematic review [38]–[41].

It is important from a health policy perspective to recognize the role of CR and education in the etiology and progression of dementia. These personal attributes are amenable to change. As a prevention strategy Ritchie et al [42] suggests that increasing a population’s ability to use skills, knowledge, and experience (crystallized intelligence) as well as increasing vegetable consumption, and eliminating depression and diabetes would have a greater impact on the prevalence and incidence of dementia than modifying known genetic risk factors.

## Supporting Information

Checklist S1 **PRISMA 2009.**
(DOC)

Appendix S1 **Data References: Meta Analysis & Qualitative analysis.**
(DOC)
