# Supplementary material for: Education and Dementia in the Context of the Cognitive Reserve Hypothesis: A Systematic Review with Meta-Analyses and Qualitative Analyses
Source: PLoS One. 2012 Jun 4;7(6):e38268. doi: 10.1371/journal.pone.0038268 (PMC3366926; doi:10.1371/journal.pone.0038268)
Supplement: Appendix S1 — Data References: Meta Analysis & Qualitative analysis. (DOC) [file pone.0038268.s002.doc]

**Appendix S2 - Data References: Meta Analysis & Qualitative analysis**

**Meta analysis**

**Prevalent Studies**

**Alzheimer’s disease**

1. Zhang MY, Katzman R, Salmon D, Jin H, Cai GJ, et al. (1990) The Prevalence of Dementia and Alzheimer's Disease in Shanghai, China: Impact of Age, Gender, and Education. Ann Neurol 27: 428-437.
2. Fratiglioni L, Grut M, Forsell Y, Viitanen M, Grafstrom M, et al. (1991) Prevalence of Alzheimer's disease and other dementias in an elderly urban population: Relationship with age, sex, and education. Neurology 141: 1886-1892.
3. The Canadian Study of Health and Aging Working Group (1994) The Canadian Study of Health and Aging: Risk factors for Alzheimer's disease in Canada. Neurology 144: 2073-2080.
4. Kondo K, Niino M, Shido K (1994) A Case-Control Study of Alzheimer's Disease in Japan – Significance of Life-Styles. Dementia 5: 314-326.
5. Graves AB, Larson EB, Edland SD, Bowen JD, McCormick WC, et al. (1994) Prevalence of Dementia and Its Subtypes in the Japanese American Population of King County, Washington State. Amer J Epidemiol 144(8): 760-771.
6. Lobo A, Saz P, Marcos G, Día JL, De-la-Cámara C (1995) The Prevalence of Dementia and Depression in the Elderly Community in a Southern European Population: The Zaragoza Study. Arch Gen Psychiatry 52(6): 497-506.
7. Liu HC, Lin KN, Teng EL, Wang SJ, Fuh JL, et al. (1995) Prevalence and Subtypes of Dementia in Taiwan: A Community Survey of 5297 Individuals. J Am Geriatr Soc 43: 144-149.
8. Mortel KF, Meyer JS, Herod B, Thornby J (1995) Education and Occupation as Risk Factors for Dementias of the Alzheimer and Ischemic Vascular Types. Dementia 6(1): 55-62.
9. Ott A, Breteler MM, van Harskamp F, Claus JJ, van der Cammen TJ, et al. (1995) Prevalence of Alzheimer's disease and vascular dementia: association with education: The Rotterdam study. BMJ 310: 970-973.
10. Callahan CM, Hall KS, Hui SL, Musick BS, Unverzagt FW, et al. (1996) Relationship of Age, Education, and Occupation With Dementia Among a Community-Based Sample of African Americans. Arch Neurol 53(2): 134-140.
11. Prencipe M, Cassini AR, Ferretti C, Lattanzio MT, Fiorelli M, et al. (1996) Prevalence of dementia in an elderly rural population: effects of age, sex, and education. J Neurol Neurosurg Psychiatry 60: 628-633.
12. Tsolaki M, Fountoulakis K, Chantzi E, Kazis A (1997) Risk Factors for Clinically Diagnosed Alzheimer’s Disease: A Case-Control Study of a Greek Population. Int Psychogeriatr 9(3): 327-341.
13. Liu HC, Fuh JL, Wang SJ, Liu CY, Larson EB, et al. (1998) Prevalence and Subtypes of Dementia in a Rural Chinese Population. Alzheimer Dis Assoc Disord 12(3): 127-134.
14. Lin RY, Lai CL, Tai CT, Liu C-, Yen YY, et al. (1998) Prevalence and subtypes of dementia in southern Taiwan: Impact of age, sex, education, and urbanization. J Neurol Sci 160: 67-75.
15. De Ronchi D, Fratiglioni L, Rucci P, Paternico A, Graziani S, et al. (1998) The effect of education on dementia occurrence in an Italian population with middle to high socioeconomic status. Neurology 50: 1231-1238.
16. Hall K, Gureje O, Gao S, Ogunniyi A, Hui SL, et al. (1998) Risk factors and Alzheimer's disease: a comparative study of two communities. Aust N Z J Psychiatry 32: 698-706.
17. Harwood DG, Barker WW, Loewenstein DA, Ownby RL, St George-Hyslop P, et al. (1999) A cross-ethnic analysis of risk factors for AD in white Hispanics and white non-Hispanics. Neurology 52:551-556.
18. Hall KS, Gao S, Unverzagt FW, Hendrie HC (2000) Low education and childhood rural residence: Risk for Alzheimer's disease in African Americans. Neurology 54(1): 95-99.
19. Bowirrat A, Treves TA, Friedland RP, Korczyn AD (2001) Prevalence of Alzheimer's type dementia in an elderly Arab population. Eur J Neurol 8: 119-23.
20. Gatz M, Svedberg P, Pedersen NL, Mortimer JA, Berg S, et al. (2001) Education and the Risk of Alzheimer's Disease: Findings From the Study of Dementia in Swedish Twins. J Gerontol A Biol Sci Med Sci 56B: 292-300.
21. Bowirrat A, Friedland RP, Farrer L, Baldwin C, Korczyn A (2002) Genetic and Environmental Risk Factors for Alzheimer's Disease in Israeli Arabs. J Mol Neurosci 19(1-2): 239-245.
22. Ravaglia G, Forti P, Maioli F, Sacchetti L, Mariani E, et al. (2002) Education, Occupation, and Prevalence of Dementia: Findings from the Conselice Study. Dement Geriatr Cogn Disord 14: 90-100.
23. Lindsay J, Laurin D, Verreault R, Hébert R, Helliwell B, et al. (2002) Risk Factors for Alzheimer's Disease: A Prospective Analysis from the Canadian Study of Health and Aging. Amer J Epidemiol 156: 445-453.
24. Mortimer JA, Snowden DA, Warkesber WR (2003) Head Circumference, Education and Risk of Dementia: Findings from the Nun Study. J Clin Exp Neuropsychol 25: 671-679.
25. Harmanci H, Emre M, Gurvit H, Bilgic B, Hanagasi H, et al. (2003) Risk Factors for Alzheimer Disease: A Population-Based Case-Control Study in Istanbul, Turkey. Alzheimer Dis Assoc Disord 17: 139-145.
26. Seidler A, Bernhardt T, Nienhaus A, Frolich L (2003) Association between the psychosocial network and dementia - a case-control study. J Psychiatr Res 37: 89-98.
27. Yu BC, Ouyang LS, Pan ZG, Wei SX, Wang YM, et al. (2004) Prevalence of dementia and its major subtypes in elderly veterans. Chinese Journal of Clinical Rehabilitation 8: 3162-3163.
28. Gatz M, Mortimer JA, Fratiglioni L, Johansson B, Berg S, Reynolds CA et al. (2006) Potentially modifiable risk factors for dementia in identical twins. Alzheimer Dement 2: 110-117.
29. Zhang ZX, Zahner GE, Román GC, Liu XH, Wu CB, et al. (2006) Socio-Demographic Variation of Dementia Subtypes in China: Methodology and Results of a Prevalence Study in Beijing, Chengdu, Shanghai, and Xian. Neuroepidemiology 27: 177-187.
30. Zhou DF, Wu CS, Qi H, Fan JH, Sun XD, et al. (2006) Prevalence of dementia in rural China: impact of age, gender and education. Acta Neurol Scand 114: 273-280.
31. Park MH, Jo SA, Jo I, Kim E, Woo EK, et al. (2008) Awareness of putative risk factors for Alzheimer’s disease among elderly Koreans. Acta Neuropsychiatrica 20: 20-24.
32. Sahadevan S, Saw SM, Gao W, Tan LC, Chin JJ, et al. (2008) Ethnic Differences in Singapore’s Dementia Prevalence: The Stroke, Parkinson’s Disease, Epilepsy, and Dementia in Singapore Study. J Am Geriatr Soc 56: 2061-2068.
33. Fischer P, Zehetmayer S, Jungwirth S, Weissgram S, Krampla W, et al. (2008) Risk Factors for Alzheimer Dementia in a Community-Based Birth Cohort at the Age of 75 Years. Dement Geriatr Cogn Disord 25: 501-507.
34. Grunblatt E, Zehetmayer S, Bartl J, Loffler C, Wichart I, et al. (2009) Genetic risk factors and markers for Alzheimer’s disease and/or depression in the VITA study. J Psychiatr Res 43: 298-308.
35. Gavrila D, Antunez C, Tormo MJ, Carles R, Garcıa Santos JM, et al. (2009) Prevalence of dementia and cognitive impairment in Southeastern Spain: the Ariadna study. Acta Neurol Scand 120: 300–307.
36. Israeli-Korn SD, Masarwa M, Schechtman E, Abuful A, Strugatsky R, et al. (2010) Hypertension increases the probability of Alzheimer's disease and of mild cognitive impairment in an Arab community in northern Israel. Neuroepidemiology 34: 99-105.
37. Mathuranath PS, Cherian PJ, Mathew R, Kumar S, George A, et al. (2010) Dementia in Kerala, South India: prevalence and influence of age, education and gender. Int J Geriatr Psychiatry 25: 290-297.

**Vascular dementia**

All duplications

**Dementia in general**

1. Liu HC, Chou P, Lin KN, Wang SJ, Fuh JL, et al. (1994) Assessing cognitive abilities and dementia in a predominantly illiterate population of older individuals in Kinmen. Psychol Med 24: 763-770.
2. Schmand B, Smit J, Lindeboom J, Smits C, Hooijer C, et al. (1997) Low Education Is a Genuine Risk Factor for Accelerated Memory Decline and Dementia. J Clin Epidemiol 50(9): 1025-1033.
3. Herrera E Jr, Caramelli P, Silvia A, Barreiros S, Nitrini R (2002) Epidemiologic Survey of Dementia in a Community-Dwelling Brazilian Population. Alzheimer Dis Assoc Disord 16(2): 103-108.
4. Zou K, Qi J, Zhang S, Guan L, He Y (2003) A cross-sectional study of senile dementia in Lianglukou street of Chongqing. Chinese Journal of Clinical Rehabilitation 7(19): 1550-1551.
5. Kahana E, Galper Y, Zilber N, Korczyn AD (2003) Epidemiology of dementia in Ashkelon: The influence of education. J Neurol 250: 424-428.
6. Ampuero I, Ros R, Royuela A, Abraira V, del Ser T, et al. (2008) Risk Factors for Dementia of Alzheimer Type and Aging-Associated Cognitive Decline in a Spanish Population Based Sample, and in Brains with Pathology Confirmed Alzheimer’s Disease. J Alzheimers Dis 14: 179-191.
7. Llibre Rodríguez J, Valhuerdi A, Sanchez II, Reyna C, Guerra MA, et al. (2008) The Prevalence, Correlates and Impact of Dementia in Cuba: A 10/66 Group Population-Based Survey. Neuroepidemiology 31: 243–251.
8. Yamada M, Mimori Y, Kasagi F, Miyachi T, Ohshita T, et al. (2009) Incidence and risks of dementia in Japanese women: Radiation Effects Research Foundation Adult Health Study. J Neurol Sci 283: 57-61.
9. Bickel H, Kurz A (2009) Education, Occupation, and Dementia: The Bavarian School Sisters Study. Dement Geriatr Cogn Disord 27: 548-556.
10. Arslantas D, Özbabalık D, Metintas S, Özkan S, Kalyoncu C, et al. (2009) Prevalence of dementia and associated risk factors in Middle Anatolia, Turkey. J Clin Neurosci 16: 1455-1459.
11. Nunes B, Silva RD, Cruz VT, Roriz JM, Pais J, et al. (2010) Prevalence and pattern of cognitive impairment in rural and urban populations from Northern Portugal. BMC Neurol 10: 42-54.
12. Saldanha D, Mani MR, Srivastava K, Goyal S, Bhattacharya D (2010) An epidemiological study of dementia under the aegis of mental health program, Maharashtra, Pune chapter. Indian J Psychiatry 52: 131-139.
13. Yaffe K, Middleton LE, Lui LY, Spira AP, Stone K, et al. (2011) Mild Cognitive Impairment, Dementia, and Their Subtypes in Oldest Old Women. Arch Neurol 68: 631-636.

**Incident Studies**

**Alzheimer’s disease**

1. Beard CM, Kokmen E, Offord KP, Kurland LT (1992) Lack of association between Alzheimer's disease and education, occupation, marital status, or living arrangement. Neurology 42: 2063-2068.
2. Stern Y, Gurland B, Tatemichi TK, Tang MX, Wilder D, et al. (1994) Influence of Education and Occupation on the Incidence of Alzheimer's Disease. JAMA 271: 1004-1010.
3. Cobb JL, Wolf PA, Au R, White R, D'Agostino RB (1995) The effect of education on the incidence of dementia and Alzheimer's in the Framingham Study. Neurology 45:1707-1712.
4. [Evans DA](http://www.ncbi.nlm.nih.gov/sites/entrez?Db=pubmed&Cmd=Search&Term="Evans DA"%5BAuthor%5D&itool=EntrezSystem2.PEntrez.Pubmed.Pubmed_ResultsPanel.Pubmed_DiscoveryPanel.Pubmed_RVAbstractPlus), [Hebert LE](http://www.ncbi.nlm.nih.gov/sites/entrez?Db=pubmed&Cmd=Search&Term="Hebert LE"%5BAuthor%5D&itool=EntrezSystem2.PEntrez.Pubmed.Pubmed_ResultsPanel.Pubmed_DiscoveryPanel.Pubmed_RVAbstractPlus), [Beckett LA](http://www.ncbi.nlm.nih.gov/sites/entrez?Db=pubmed&Cmd=Search&Term="Beckett LA"%5BAuthor%5D&itool=EntrezSystem2.PEntrez.Pubmed.Pubmed_ResultsPanel.Pubmed_DiscoveryPanel.Pubmed_RVAbstractPlus), [Scherr PA](http://www.ncbi.nlm.nih.gov/sites/entrez?Db=pubmed&Cmd=Search&Term="Scherr PA"%5BAuthor%5D&itool=EntrezSystem2.PEntrez.Pubmed.Pubmed_ResultsPanel.Pubmed_DiscoveryPanel.Pubmed_RVAbstractPlus), [Albert MS](http://www.ncbi.nlm.nih.gov/sites/entrez?Db=pubmed&Cmd=Search&Term="Albert MS"%5BAuthor%5D&itool=EntrezSystem2.PEntrez.Pubmed.Pubmed_ResultsPanel.Pubmed_DiscoveryPanel.Pubmed_RVAbstractPlus), et al. (1997) Education and Other Measures of Socioeconomic Status and Risk of Incident Alzheimer Disease in a Defined Population of Older Persons. Arch Neurol 54: 1399-405.
5. Zhang M, Katzman R, Yu E, Liu W, Xiao SF, et al. (1998) A preliminary analysis of incidence of dementia in Shanghai, China. Psychiatry Clin Neurosci 52: S291-294.
6. Geerlings MI, Schmand B, Jonker C, Lindeboom J, Bouter LM (1999) Education and incident Alzheimer's disease: a biased association due to selective attrition and use of a two-step diagnostic procedure? Int J Epidemiol 28: 492-497.
7. Launer LJ, Andersen K, Dewey ME, Letenneur L, Ott A, et al. (1999) Rates and risk factors for dementia and Alzheimer's disease: Results from EURODEM pooled analyses. Neurology 52: 78-84.
8. Letenneur L, Launer LJ, Andersen K, Dewey ME, Ott A, et al. (2000) Education and the Risk for Alzheimer's Disease: Sex Makes a Difference: EURODEM Pooled Analyses. Amer J Epidemiol 151: 1064-1071.
9. He YL, Zhang XK, Zhang MY (2000) Psychosocial risk factors for Alzheimers’s Disease. Hong Kong Journal of Psychiatry 10: 2-7.
10. Kawas C, Gray S, Brookmeyer R, Fozard J, Zonderman A (2000) Age-specific incidence rates of Alzheimer's disease: The Baltimore Longitudinal Study of Aging. Neurology 54: 2072-2077.
11. Qiu C, Backman L, Winblad B, Aguero-Torres H, Fratiglioni L (2001) The Influence of Education on Clinically Diagnosed Dementia Incidence and Mortality Data From the Kungsholmen Project. Arch Neurol 58: 2034-2039.
12. Karp A, Kareholt I, Qiu C, Bellander T, Winblad B, et al. (2004) Relation of Education and Occupation-based Socioeconomic Status to Incident Alzheimer's Disease. Am J Epidemiol 159: 175-183.
13. Yip AG, Bayne C, Matthews FE, MRC Cognitive Function and Ageing Study (2006) Risk factors for incident dementia in England and Wales: The Medical Research Council Cognitive Function and Ageing Study. A population-based nested case-control study. Age Ageing 35: 154-160.
14. McDowell I, Xi G, Lindsay J, Tierney M (2007) Mapping the connections between education and dementia. J Clin Exp Neuropsychol 29: 127-141.

**Vascular dementia**

1. Lindsay J, Hebert R, Rockwood K (1997) The Canadian Study of Health and Aging Risk Factors for Vascular Dementia. Stroke 28: 526-530.
2. Yang H, Li J, Zhou H (2007) Logistic regression analysis on risk factors for vascular dementia following cerebral infarction in 403 patients from Chongqing City. Neural Regeneration Research 2: 360-364.

**Dementia in general**

1. Solfrizzi V, Panza F, Colacicco AM, D'Introno A, Capurso C, et al. (2004) Italian Longitudinal Study on Aging Working Group. Vascular risk factors, incidence of MCI, and rates of progression to dementia. Neurology 63: 1882-1891.
2. Ngandu T, von Strauss E, Helkala EL, Winblad B, Nissinen A, et al. (2007) Education and dementia: What lies behind the association? Neurology 69: 1442-1450.
3. Rusanen M, Kivipelto M, Quesenberry CP, Zhou J, Whitmer RA (2011) Heavy Smoking in Midlife and Long-term Risk of Alzheimer Disease and Vascular Dementia. Arch Intern Med 171: 333-339.

**Qualitative analyses**

**Prevalence**

1. Letenneur L, Gilleron V, Commenges D, Helmer C, Orgogozo JM, et al. (1999) Are sex and educational level independent predictors of dementia and Alzheimer's disease? Incidence data from the PAQUID project. J Neurol Neurosurg Psychiatry 66: 177-183.
2. [Yamada M](http://www.ncbi.nlm.nih.gov/sites/entrez?Db=pubmed&Cmd=Search&Term="Yamada M"%5BAuthor%5D&itool=EntrezSystem2.PEntrez.Pubmed.Pubmed_ResultsPanel.Pubmed_DiscoveryPanel.Pubmed_RVAbstractPlus), [Sasaki H](http://www.ncbi.nlm.nih.gov/sites/entrez?Db=pubmed&Cmd=Search&Term="Sasaki H"%5BAuthor%5D&itool=EntrezSystem2.PEntrez.Pubmed.Pubmed_ResultsPanel.Pubmed_DiscoveryPanel.Pubmed_RVAbstractPlus), [Mimori Y](http://www.ncbi.nlm.nih.gov/sites/entrez?Db=pubmed&Cmd=Search&Term="Mimori Y"%5BAuthor%5D&itool=EntrezSystem2.PEntrez.Pubmed.Pubmed_ResultsPanel.Pubmed_DiscoveryPanel.Pubmed_RVAbstractPlus), [Kasagi F](http://www.ncbi.nlm.nih.gov/sites/entrez?Db=pubmed&Cmd=Search&Term="Kasagi F"%5BAuthor%5D&itool=EntrezSystem2.PEntrez.Pubmed.Pubmed_ResultsPanel.Pubmed_DiscoveryPanel.Pubmed_RVAbstractPlus), [Sudoh S](http://www.ncbi.nlm.nih.gov/sites/entrez?Db=pubmed&Cmd=Search&Term="Sudoh S"%5BAuthor%5D&itool=EntrezSystem2.PEntrez.Pubmed.Pubmed_ResultsPanel.Pubmed_DiscoveryPanel.Pubmed_RVAbstractPlus), et al. (1999) Prevalence and Risks of Dementia in the Japanese Population: REFR's Adult Health Study Hiroshima Subjects. J Am Geriatr Soc 47: 189-195.
3. Tyas SL, Manfreda J, Strain L, Montgomery PR (2001) Risk factors for Alzheimer's disease: a population-based, longitudinal study in Manitoba, Canada. Int J Epidemiol 30: 590-597.
4. Anttila T, Helkala EL, Kivipelto M, Hallikainen M, Alhainen K, et al. (2002) Midlife income, occupation, APOE status, and dementia. Neurology 59: 887-893.
5. Di Carlo A, Baldereschi M, Amaducci L, Lepore V, Bracco L, et al. (2002) Incidence of Dementia, Alzheimer's Disease, and Vascular Dementia in Italy. The ILSA Study. J Am Geriatr Soc 50: 41-48.
6. Miech RA, Breitner JC, Zandi PP, Khachaturian AS, Anthony JC, et al. (2002) Incidence of AD may decline in the early 90s for men, later for women. Neurology 58: 209-218.
7. Wilson RS, Bennett DA, Bienias JL, Aggarwal NT, Mendes De Leon CF, et al. (2002) Cognitive activity and incident AD in a population-based sample of older persons. Neurology 59: 1910-1914.
8. Yamada M, Kasagi F, Sasaki H, Masunari N, Mimori Y, et al. (2003) Association Between Dementia and Midlife Risk Factors: the Radiation Effects Research Foundation Adult Health Study. J Am Geriatr Soc; 51:410-414.
9. Ravaglia G, Forti P, Maioli F, Martelli M, Servadei L, et al. (2005) Incidence and etiology of dementia in a large elderly Italian population. Neurology 64: 1525-1530.
10. Tognoni G, Ceravolo R, Nucciarone B, Bianchi F, Dell'Agnello G, et al. (2005) From mild cognitive impairment to dementia: a prevalence study in a district of Tuscany, Italy. Acta Neurol Scand 112: 65-71.
11. Lin JC, Hsu WC, Hsu HP, Fung HC, Chen ST (2006) Risk Factors for Vascular Dementia: A Hospital-Based Study in Taiwan. Acta Neurol Taiwan 17: 22-6.
12. Shadlen MF, Sisovick D, Fitzpatrick AL, Dulberg C, Kuller LH, et al. (2006) Education, Cognitive Test Scores, and Black-White Differences in Dementia Risk. J Am Geriatr Soc 54: 898-905.
13. van Oijen M, de Jong FJ, Hofman A, Koudstaal PJ, Breteler MM (2007) Subjective memory complaints, education, and risk of Alzheimer' s disease. Alzheimer's & Dementia 3: 92-97.
14. Galasko D, Salmon D, Gamst A, Olichney J, Thal LJ, et al. (2007) Prevalence of dementia in Chamorros on Guam: relationship to age, gender, education, and APOE. Neurology 68: 1772-1781.
15. Lee JY, Chang SM, Jang HS, Chang JS, Suh GH, et al. (2008) Illiteracy and the incidence of Alzheimer’s disease in the Yonchon County survey, Korea. Int Psychogeriatr 20: 976-985.
16. Yamada M, Mimori Y, Kasagi F, Miyachi T, Ohshita T, et al. (2008) Incidence of Dementia, Alzheimer Disease, and Vascular Dementia in a Japanese Population: Radiation Effects Research Foundation Adult Health Study. Neuroepidemiology; 30: 152-160.
17. Hebert LE, Bienias JL, Aggarwal NT, Wilson RS, Bennett DA, et al. (2010) Change in risk of Alzheimer disease over time. Neurology 75: 786–91.
18. EClipSE Collaborative Members (2010) Education, the brain and dementia: neuroprotection or compensation? Brain 133: 2210-2216.
19. Rastas S, Pirttila T, Mattila K, Verkkoniemi A, Juva K, et al. (2010) Vascular risk factors and dementia in the general population aged >85 years Prospective population-based study. Neurobiol Aging 31: 1-7.
20. Kim KW, Park JH, Kim MH, Kim MD, Kim BJ, et al. (2011) A Nationwide Survey on the Prevalence of Dementia and Mild Cognitive Impairment in South Korea. J Alzheimers Dis 23: 281-291.

**Cognitive decline**

1. Filley CM, Brownell HH, Albert ML (1985) Education provides no protection against Alzheimer's disease. Neurology 35: 1781-1784.
2. Teri L, McCurry SM, Edland SD, Kukull WA, Larson EB (1995) Cognitive Decline in Alzheimer's Disease: A Longitudinal Investigation of Risk Factors for Accelerated Decline. J Gerontol A Biol Sci Med Sci 50A: M49-55.
3. Small BJ, Viitanen M, Winblad B, Backman L (1997) Cognitive Changes in Very Old Persons with Dementia: The Influence of Demographic, Psychometric, and Biological Variables. J Clin Exp Neuropsychol 19(2): 245-260.
4. Aguero-Torres H, Fragiglioni L, Guo Z, Viitanen M, Winblad B (1998) Prognostic Factors in Very Old Demented Adults: A Seven-Year Follow-Up From a Population-Based Survey in Stockholm. J Am Geriatr Soc 46: 444-452.
5. Weiner MF, Edland SD, Kukull WA, Risser RC (1998) No protective effect of education in Alzheimer's disease: Findings from the CERAD database. Alzheimer's Research 4: 1-4.
6. Stern Y, Albert S, Tang MX, Tsai WY (1999) Rate of Memory Decline in AD is related to education and occupation: Cognitive Reserve? Neurology 53: 1942-1947.
7. Wilson RS, Bennett DA, Gilley DW, Beckett LA, Barnes LL, et al. (2000) Premorbid Reading Activity and Patterns of Cognitive Decline in Alzheimer Disease. Arch Neurol 57: 1718-1723.
8. Fritsch T, McClendon MJ, Smyth KA, Ogrocki PK (2002) Effects of Educational Attainment and Occupational Status on Cognitive and Functional Decline in Persons With Alzheimer-Type Dementia. Int Psychogeriatr 14: 347-363.
9. Tuokko H, Garrett DD, McDowell I, Silverberg N, Kristjansson B (2003) Cognitive decline in high-functioning older adults: reserve or ascertainment bias? Aging Ment Health 7: 259-270.
10. Suh GH, Ju YS, Yeon BK, Shah A (2004) A longitudinal study of Alzheimer’s disease: rates of cognitive and functional decline. Int J Geriatr Psychiatry 19: 817-824.
11. Tervo S, Kivipelto M, Hänninen T, Vanhanen M, Hallikainen M, et al. (2004) Incidence and Risk Factors for Mild Cognitive Impairment: A Population-Based Three-Year Follow-Up Study of Cognitively Healthy Elderly Subjects. Dement Geriatr Cogn Disord 17: 196-203.
12. Wilson RS, Li Y, Aggarwal MD, Barnes LL, McCann JJ, et al. (2004) Education and the course of cognitive decline in Alzheimer disease. Neurology 63: 1198-1202.
13. Le Carret N, Auriacombe S, Letenneur L, Bergua V, Dartigues JF, et al. (2005) Influence of education on the pattern of cognitive deterioration in AD patients: The cognitive reserve hypothesis. Brain and Cognition 57: 120-126.
14. Andel R, Vigen C, Mack WJ, Clark LJ, Gatz M (2006) The effect of education and occupational complexity on rate of cognitive decline in Alzheimer’s patients. Int Neuropsychol Soc 12: 147-152.
15. Hall CB, Debry C, LeValley A, Katz MJ, Verghese J, et al. (2007) Education delays accelerated decline on a memory test in persons who develop dementia. Neurology 69: 1657-1664.
16. Bruandet A, Richard F, Bombois S, Maurage CA, Masse I, et al. (2008) Cognitive Decline and Survival in Alzheimer’s Disease according to Education Level. Dement Geriatr Cogn Disord 25: 74-80.
17. Helzner EP, Luchsinger JA, Scarmeas N, Cosentino S, Brickman AM, et al. (2009) Contribution of Vascular Risk Factors to the Progression in Alzheimer Disease. Arch Neurol 66: 343-348.
18. Musicco M, Palmer K, Salamone G, Lupo F, Perri R, et al. (2009) Predictors of progression of cognitive decline in Alzheimer’s disease: the role of vascular and sociodemographic factors. J Neurol 256: 1288–1295.
19. Chaves ML, Camozzato AL, Kohler C, Kaye J (2010) Predictors of the Progression of Dementia Severity in Brazilian Patients with Alzheimer’s Disease and Vascular Dementia. Int J Alzheimers Dis. doi:10.4061/2010/673581.

**Age of onset**

1. Montz DJ, Petitti DB (1993) Association of Education with Reported Age of Onset and Severity of Alzheimer's Disease at Presentation: Implications for the Use of Clinical Samples. Am J Epidemiol 137: 456-462.
2. Pai MC, Hsiao S (2002) Incipient Symptoms of Alzheimer's Disease and Effect of Education on the onset Age: A Study of 155 Taiwanese Patients. Acta Neurol Taiwan 11: 66-69.
3. Roe CM, Xiong C, Grant E, Miller JP, Morris JC (2008) Education and Reported Onset of Symptoms Among Individuals with Alzheimer Disease. Arch Neurol 65: 108-111.
4. Lupton MK, Stahl D, Archer N, Foy C, Poppe M, et al. (2010) Education, occupation and retirement age effects on the age of onset of Alzheimer’s disease. Int J Geriatr Psychiatry 25: 30-36.

**Mortality**

1. Stern Y, Tang MX, Denaro J, Mayeux R (1995) Increased Risk of Mortality in Alzheimer's Disease Patients with More Advanced Educational and Occupational Attainment. Ann Neurol 37: 590-595.
2. Geerlings MI, Deeg DJ, Schmand B, Lindeboom J, Jonker C (1997) Increased risk of mortality in Alzheimer's disease patients with higher education? A replication study. Neurology 49: 798-802.
3. Brehaut JC, Raina P, Lindsay J (2004) Does cognitive status modify the relationship between education and mortality? Evidence from the Canadian Study of Health and Aging. Int Psychogeriatr 16: 75-91.
4. Pavlik VN, Doody RS, Massman PJ, Chan W (2006) Influence of Premorbid IQ and Education on Progression of Alzheimer's Disease. Dement Geriatr Cogn Disord 22: 367-377.

**Clinical Performance**

1. Swanwick GR, Coen RF, Maguire CP, Kirby M, Walsh JB, et al. (1999) The association between demographic factors, disease severity and the duration of symptoms at clinical presentation in elderly people with dementia. Age Ageing 28: 295-299.
2. Ott BR, Heindel WC, Papandonatos GD, Festa EK, Davis JD, et al. (2008) A longitudinal study of drivers with Alzheimer disease. Neurology 70: 1171-1178.
3. Aguera-Ortiz L, Frank-Garcıa A, Gil P, Moreno A, 5E Study Group (2010) Clinical progression of moderate-to-severe Alzheimer’s disease and caregiver burden: a 12-month multicenter prospective observational study. Int Psychogeriatr 22: 1265-1279.

**Pathology and Imaging**

1. Stern Y, Alexander GE, Prohovnik I, Mayeux R (1992) Inverse Relationship Between Education and Parietotemporal Perfusion Deficit in Alzheimer's Disease. Ann Neurology 32: 371-375.
2. Kidron D, Black SE, Stanchev P, Buck B, Szakai JP, et al. (1997) Quantitative MR volumetry in Alzheimer’s disease: Topograpic markers and the effects of sex and education. Neurology 49: 1504-1512.
3. Bowler JV, Munoz DG, Merskey H, Hachinski V (1998) Factors affecting the age of onet and rate of progression of Alzheimer’s disease. J Neurol Neurosurg Psychiatry 65:184-190.
4. Del Ser T, Hachinski V, Mersky H, Munoz DG (1999) An autopsy-verified study of the effect of education on degenerative dementia. Brain 122: 2309-2319.
5. Zubenko GS, WinWood E, Jacobs B, Teply I, Stiffler JS, et al. (1999) Prospective Study of Risk Factors for Alzheimer’s Disease: Results at 7.5 Years. Am J Psychiatry 156: 50-57.
6. Munoz DG, Ganapathy GR, Eliasziw M, Hachinski V (2000) Educational Attainment and Socioeconomic Status of Patients With Autopsy-Confirmed Alzheimer Disease. Arch Neurol 57: 85-89.
7. Bennett DA, Wilson RS, Schneider JA, Evans DA, de Leon M, et al. (2003) Education modifies the relation of AD pathology to level of cognitive function in older persons. Neurology 60: 1909-1915.
8. Liao YC, Liu RS, Teng EL, Lee YC, Wang PN, et al. (2005) Cognitive Reserve: A SPECT Study of 132 Alzheimer's Disease Patients with an Education Range of 0-19 Years. Dement Geriatr Cogn Disord 20: 8-14.
9. Mortimer JA, Boresstein AR, Goshe KM, Snowden DA (2005) Very early detection of Alzheimer Neuropathology and the Role of Brain Reserve in Modifying Its Clinical Expression. J Geriatr Psychiatry Neurol 18: 218-223.
10. Perneczky R, Drzezga A, Diehl-Schmid J, Schmid G, Wohlschläger A , et al. (2006) Schooling mediates brain reserve in Alzheimer’s disease: findings of fluoror-deoxy-glucose-positron emission tomography. J Neurol Neurosurg Psychiatr 77: 1060-1063
11. Roe CM, Xiong C, Miller JP, Morris JC (2007) Education and Alzheimer disease without dementia: support for the cognitive reserve hypothesis. Neurology 68: 223-228.
12. Hanyu H, Sato T, Shimizu S, Kanetaka H, Iwamoto T, et al. (2008) The effect of education on rCBF changes in Alzheimer’s disease: a longitudinal SPECT study. Eur J NucL Med Mol Imaging 35: 2182-2190.
13. Koepsell TD, Kurland BE, Harel O, Johnson EA, Zhou XH, et al. (2008) Education, cognitive function, and severity of neuropathology in Alzheimer disease. Neurology 70: 1732-1739.
14. Cordonnier C, Leys D, Dumont F, Deramecourt V, Bordet R, et al. (2010) What are the causes of pre-existing dementia in patients with intracerebral haemorrhages? Brain 133: 3281-3289.
